# MUC4 and MMP7 in saliva and gingival crevicular fluid (GCF) in adolescents at West Bengal, India

**DOI:** 10.6026/97320630018165

**Published:** 2022-03-31

**Authors:** Sayan Chattopadhyay, Rachita Arora, Amit Kishor, Shivani Singh, Abhijeet Alok, Mainak Mitra

**Affiliations:** 1 Burdwan Dental College and Hospital, West Bengal, India; 2 Primary Health Centre Baniyapur, Chapra, Bihar, India; 3 Dr B R Ambedkar Institute of Dental Sciences and Hospital, Patna, Bihar, India; 4 Primary Health Centre, Khaira, Jamui, Bihar, India

**Keywords:** Gingival Crevicular Fluid (GCF), matrix metalloproteinase 7(MMP7), mucin 4(MUC4), periodontitis, saliva

## Abstract

Mucin 4 (MUC4) and matrix metalloproteinase 7 (MMP7) have been reported to be associated with chronic periodontitis as seen in gingival tissue biopsies. Therefore, it is of interest to estimate the levels of MUC4 and MMP7 in saliva and gingival crevicular fluid (GCF) samples of periodontitis in adolescents patients at West Bengal, India. MUC4 levels were significantly lower in saliva and GCF from periodontitis patients compared to healthy controls. However, MMP7 levels were found to be significantly higher in saliva and GCF from periodontitis patients. Thus, MUC4 and MMP7 are biomarkers for periodontitis diagnosis and towards further consideration.

## Background:

Periodontitis is chronic inflammatory disease of supporting tooth structure that affects 9-15% of the adult population worldwide [1]. It often involves loss of alveolar bone and, if left uncured, tooth loss might also occur [2]. Initiation occurs by formation of microbial biofilm on the teeth, in vicinity of the gingival tissue. Microbial products such as lipo-poly-saccharides (LPS) infiltrate the gingival tissue, triggering an initial immune-inflammatory response [1,2]. This leads to release of inflammatory mediators (chemicals) such as cytokines, prostaglandins (Pgs), and reactive oxygen species (ROS), as well as proteolytic enzymes such as matrix metallo-proteinases (MMPs) [1-3]. Mucins play a core role in innate immunity by promotion of aggregation and removal of microbes from the oral cavity [4]. Salivary film is composed of a gel-forming constituent known as mucins covering the epithelium, serving as a diffusion membrane against antigens or foreign substances [4]. Around 20 members belong to mucin family that can be further divided as secreted mucins that modulate other salivary proteins and cell surface mucins [4]. Secreted mucins also interact with microbes to facilitate their clearance and lower their pathogenicity [4]. Microbial products and toxins activate proinflammatory cytokines that in turnstimulate up-regulation of proteolytic enzymes, including MMPs. Structurally related but genetically distinct MMPs are the leading proteins causing remodelling of extra-cellular matrix contributing to periodontal destruction and aiding in pathogenesis of periodontitis [5,6] MMP8 and MMP9 are found elevated in periodontitis patients seen in saliva and GCF samples of these patients [5,6] The MMPs activity is controlled at level of gene expression through pro-enzymes activation, and by inhibition by tissue inhibitors of matrix metallo-proteinases (TIMPs) referred to as specific endogenous inhibitors [5,6] A balance exists between MMPs and TIMPs and any changes in that ratio is suggestive of periodontal tissue breakdown. MMP activity thus regulates homeostasis maintaining a constant internal environment [5,6]. Many studies have recently reported that MUC4 and MMP7 are expressed differentially in biopsies of gingival tissue taken from periodontitis patients and healthy controls investigated through RNA sequencing [5-8]. Therefore, it is of interest to evaluate the levels of Mucin 4 and matrix metallo-proteinases 7 in saliva and GCF samples [9,10] taken from periodontitis patients and healthy controls in adolescents' patients at West Bengal, India.

## Materials and methods:

Collection and preparation of saliva samples after taking written informed consent, 40 adolescent (10-15years) subjects diagnosed with chronic periodontitis and 39 healthy controls were recruited in study showing clinical signs of periodontitis like bleeding on probing in more than 30% of sites examined, pocket depth more than 4mm with radio-graphically confirmed bone loss were included in patients group while others showing no signs of bone loss and probing pocket depth (PPD) less than 4mm were included in healthy control group. 84% of patients had (PPD) ≥ 6 mm and 16% 4-5mm respectively. Saliva samples were collected from all participants. Stimulated saliva samples were collected into test tubes by making participants chew paraffin tablets for about 5 min. Saliva samples were immediately frozen at -20°C immediately after collection until further processing. Then after thawing and centrifugation was done at 500 g for 10 min at 4°C. The supernatant layers were then aliquot into 1.5 ml Eppendorf tubes to be stored at -80°C until further quantification and analysis. Analysis of salivary levels of total protein, MUC4, and MMPs the total protein concentrations were calculated using the ELISA kitas per to the manufacturer's instructions, using bovine serum albumin as standard. The levels of MUC4 and MMP7 were calculated using commercially available ELISA kits. Prior to analysis of MUC4 and MMP7, in a ratio of 1:2 the collected saliva samples were diluted in phosphate buffered saline (PBS) and in calibrator diluent buffer (CDB) respectively. The sensitivities for the assays that were used for MMP7 was0.084 ng/ml, 0.08 ng/ml for MMP8 and 0.134 ng/ml for MUC4.

## Collection and preparation of GCF samples:

Gingival crevicular fluid samples were collected from 20 periodontitis patients. Prior to sample collection, supra-gingival plaque was cleared off the tooth surface with a cotton pellet and the surface was gently air dried. GCF samples were acquired from the buccal (mesial and distal) sites from one tooth by inserting two separate perio-paper strips into the gingival crevice until slight resistance was felt, and left for 30s. Any blood contaminated samples of GCF were discarded. Immediately after GCF collection, the paper strips were frozen at -20°C until further processing. ELISA testing was done in a similar fashion as mentioned earlier for saliva samples.

## Results:

### Subject characteristics:

The characteristics of the study participants including patients with periodontitis (n = 40) and healthy controls (n = 39) from which saliva samples were obtained are presented in Table 1(see PDF).

Multiple linear regression analyses were carried out to investigate the influence of periodontitis on the salivary levels of MUC4 and MMP7, adjusted for age and smoking habits (Table 2 - see PDF). The analyses were performed using the total concentrations of MUC4 and MMP7 as well as their concentrations related to the total protein levels. The results revealed that periodontitis contributed significantly (p < 0.05) to the levels of MUC4 in saliva samples, when adjusting for age and smoking. MUC4 remained significant (p < 0.01) also when related to the total protein concentrations in the saliva samples (Table 2 - see PDF). In addition, the analysis revealed that smoking contributed significantly to the levels of MMP7 as well as to MMP7 related to total protein concentrations (Table 2 - see PDF).

### MUC4 and MMP7 levels in GCF samples:

In addition to saliva samples, the levels of MUC4 and MMP7 were also investigated in GCF samples from a cohort of 40 subjects with periodontitis and healthy controls. The mean age of subjects was significantly (p < 0.01) higher in the periodontitis group compared to the healthy controls (67.1 ± 9.5 and 38.5 ± 10.9 years respectively). The periodontitis group, comprising 10 females and 10 males, had significantly (p < 0.01) higher PPD (mean 6.9 ± 2.0 mm) and BOP (45%), than the healthy control group, comprising 14 females and six males (PPD ≤ 3 mm, BOP 5%). In agreement with our findings in saliva, the total concentrations of MUC4 as well as MUC4 related to total protein concentration were significantly (p < 0.05 and p < 0.01 respectively) lower in the GCF from periodontitis patients relative to controls (Table S3 - see PDF). Moreover, the levels of MMP7, as well as the total protein concentrations, were significantly (p < 0.01) higher in GCF from periodontitis patients. Multiple linear regression analyses were also performed with the total concentrations of MUC4 and MMP7 as well as in relation to protein concentrations as dependent variables, adjusted for age and smoking habits (Table 3 - see PDF). The analyses revealed that periodontitis was significantly (p < 0.01) associated with the GCF levels of MUC4, and MUC4 related to total protein concentrations, as well as with the levels of MMP7 (Table 3 - see PDF).

## Discussion:

Many studies have recently reported that MUC4 and MMP7 are expressed differentially in biopsies of gingival tissue taken from periodontitis patients and healthy controls investigated through RNA sequencing [11]. Here, we report, to the best of our knowledge, very first time exploring significantly different levels of the above mentioned two proteins in saliva and GCF samples from patients with periodontitis compared to healthy controls. Furthermore, in this study group, combination of the salivary levels of MUC4 and MMP7 is also demonstrated that has shown the potential of discriminating between individuals with and without periodontitis [11]. In this study, protein levels of MUC4, determined by ELISA using specific antibodies, were significantly lower in saliva and GCF samples of patients with periodontitis as compared to healthy controls. MUC4 has previously been implicated in cancer, including pancreatic, breast, and lung. In reference to pathogenesis of periodontitis, studies have reported higher levels of mucins in general in saliva samples from periodontitis patients than in healthy subjects. These studies used, however, the Alcian blue method that's known to stain glycoproteins in general and can therefore not distinguish different mucin family members, up to date 20 members [11]. At the mRNA level, our previous sequencing study, investigating the whole transcriptome in gingival tissue biopsies from periodontitis patients and healthy controls, identified higher expression of MUC4 in gingival tissue biopsies from patients with periodontitis [11]. The contrasting findings in tissue of gingiva versus oral fluids may be due to the fact that MUC4 exists in both secreted and membrane‐bound form and that the balance between these two forms may be altered due to reprogramming of signalling pathways of the MUC4 gene in response to inflammation [11]. As a result of the biofilm formed adjacent to the gingival tissue, the membrane-bound form of MUC4 may increase in order to prevent the bacteria to access the cell surface and to protect the gingival tissue [11]. This suggestion is in line with our previous results demonstrating increased MUC4 expression in oral epithelial cells stimulated with LPS [11]. Another explanation for the decreased levels of MUC4 in saliva samples might be due to proteolytic degradation, either by enzymes up‐regulated by the host or expressed by microbes [11]. The reduced levels of MUC4 in saliva might lead to their lower capability to agglutinate as well as cleanse oral pathogens or antigens, allowing biofilm formation on the tooth surface, leading to subclinical constant inflammatory response in periodontitis patients [11]. Our findings have demonstrated higher levels of MMP7 in saliva and GCF samples from patients with periodontitis relative to healthy controls are in agreement with our RNA sequencing results, which revealed overexpression of MMP7 in gingival tissue biopsies of patients with periodontitis [10]. Levels of MMP7 have, to our knowledge, not previously been reported in saliva of periodontitis patients [10]. With regard to GCF, MMP7 has been reported to be elevated in samples from patients with adult periodontitis relative to GCF samples collected from patients with localized juvenile periodontitis and controls [10]. Another study has also no significant differences in levels of total MMP7 in GCF samples from 20 patients with different periodontal diseases and healthy controls [10]. After correcting for the volume of GCF obtained from each site, however, the levels of MMP7 were identified as significantly lower in periodontitis patients than in healthy controls [10]. Nonetheless, one limitation with this study as well as our current study is the small number of GCF samples and additional studies should be performed in order to validate these findings [11,12]. Another limitation with our study is the relatively high age of subjects in the periodontitis group relative to the healthy control group. Nevertheless, our multiple regression analyses were adjusted for age and therefore the results would be applicable to younger patients as well.

## Conclusion:

We report significantly varied levels of MUC4 and MMP7 in saliva and GCF of patients with periodontitis in comparison to healthy controls. This suggests that MUC4 and MMP7 combination are diagnostic markers for periodontitis.
